# Beyond Oncological Hyperthermia: Physically Drivable Magnetic Nanobubbles as Novel Multipurpose Theranostic Carriers in the Central Nervous System

**DOI:** 10.3390/molecules25092104

**Published:** 2020-04-30

**Authors:** Eleonora Ficiarà, Shoeb Anwar Ansari, Monica Argenziano, Luigi Cangemi, Chiara Monge, Roberta Cavalli, Federico D’Agata

**Affiliations:** 1 Department of Neurosciences, University of Turin, 10124 Turin, Italy; shoebanwarmohammedkhawja.ansari@unito.it (S.A.A.); federico.dagata@unito.it (F.D.); 2 Department of Scienza e Tecnologia del Farmaco, University of Turin, 10125 Turin, Italy; monica.argenziano@unito.it (M.A.); luigi.cangemi@unito.it (L.C.); chiara.monge@edu.unito.it (C.M.); roberta.cavalli@unito.it (R.C.)

**Keywords:** magnetic nanoparticles, nanobubbles, brain barriers, magnetic driving, brain tumors, theranostic

## Abstract

Magnetic Oxygen-Loaded Nanobubbles (MOLNBs), manufactured by adding Superparamagnetic Iron Oxide Nanoparticles (SPIONs) on the surface of polymeric nanobubbles, are investigated as theranostic carriers for delivering oxygen and chemotherapy to brain tumors. Physicochemical and cyto-toxicological properties and in vitro internalization by human brain microvascular endothelial cells as well as the motion of MOLNBs in a static magnetic field were investigated. MOLNBs are safe oxygen-loaded vectors able to overcome the brain membranes and drivable through the Central Nervous System (CNS) to deliver their cargoes to specific sites of interest. In addition, MOLNBs are monitorable either via Magnetic Resonance Imaging (MRI) or Ultrasound (US) sonography. MOLNBs can find application in targeting brain tumors since they can enhance conventional radiotherapy and deliver chemotherapy being driven by ad hoc tailored magnetic fields under MRI and/or US monitoring.

## 1. Introduction

Magnetic nanocarriers have been extensively investigated as hyperthermic agents in combination with radiotherapy to treat superficial and deep tumors [1,2,3]. In particular, Magnetic Fluid Hyperthermia (MFH) consists of the in situ administration of a stable colloidal suspension of biocompatible Superparamagnetic Iron Oxide Nanoparticles (SPIONs) which can produce endogenous heat generation following activation by external magnetic fields [4]. Clinical applications of MFH has been approved by the Food and Drug Administration (FDA) and is already marketed (MagForce ^®^ activated by MFH ^®^ 300F or NanoActivator ^®^). 

As tumor oxygenation is one of the main targets of the hyperthermic treatment, our group investigated the use of oxygen carriers “decorated” by SPIONs as hyperthermic agent [5], showing that in addition to heating (temperature increase of some °C could be reached already at low magnetic field) such Magnetic Oxygen-Loaded Nanobubbles (MOLNBs) are easily internalized by cells, do not produce toxic effects, deliver oxygen in a sustained way, and can be monitored either by using clinical Ultrasound (US) sonography and by Magnetic Resonance Imaging (MRI) [5].

In the present study we further investigated MOLNBs looking at possible applications besides hyperthermia, i.e., magnetic driving and delivery systems. 

In particular, the attention was focused on the quantitative methods for engineered nanoparticles to cross brain barriers and to effectively target the Central Nervous System (CNS) [6]. Various chemical and physical approaches have been proposed to satisfy the requirement for effective therapy and imaging [4,7].

As a matter of fact, magnetic nanoparticles have already been proposed to bypass the Blood–Brain Barrier (BBB) to treat glioblastomas and neurodegenerative diseases [8,9,10,11,12] as well as in regenerative medicine [13,14] and drug delivery [15,16]. Very recently magnetic nanoparticles have been proposed as transducers in advanced neuromodulation, via the hysteretic heat when exposed to alternated magnetic fields [17]. 

We propose MOLNBs as a new theranostic application for the treatment of cerebral tumors, based on their ability to carry oxygen (a well-known radiotherapy enhancer) and a proper load of chemotherapy drugs (doxorubicin and possibly temozolomide) [18]. Indeed, we speculate that they can be locally delivered to the Cerebro-spinal Fluid (CSF) by spinal injection, magnetically driven towards the part of the choroid plexus anatomically most proximal to the tumor mass where the drug and oxygen cargoes are delivered after crossing the barrier from the CSF to the brain interstitial fluids.

Previous imaging by Computed Tomography (CT) and/or MRI can guide the tailoring of the magnetic field required for optimal driving to the tumor, whereas post-treatment imaging by MRI and US (when the skull does not shield the target) allows the monitoring of the MOLNBs final concentration.

To assess the feasibility of this innovative delivery approach we investigated whether such nanocarriers are safe, biocompatible, not cytotoxic, and not hemolytic (in case of systemic administration or possible interactions with blood). In addition, we evaluated their internalization capability by human brain microvascular endothelial cells (hBMECs) and, finally, if we can drive the MOLNBs using proper magnetic fields, which is known to be a weakness of all the similar procedure [19]. 

Accordingly, together with a specific assessment of the physicochemical and biocompatibility properties of the MOLNBs, their response to the external magnetic field produced by a permanent magnet has been investigated via ultrasonic imaging.

## 2. Results

### 2.1. Physicochemical Characterization of MOLNBs Formulations

The physicochemical characterization of OLNBs and MOLNBs is reported in Table 1. The negative value of the zeta potential is the fingerprint of dextran sulfate on the NB surface. The average zeta potential of SPIONs was measured to be +16.24 mV. The positive charge of SPIONs is appropriate for binding with negatively charged dextran sulfate polymer shell by electrostatic interactions. After binding of SPIONs the polymer shell surface reduced its zeta potential by 45%. Other specific parameters of the OLNBs nanosuspension such as pH, viscosity, and osmolarity were 6.52, 0.98 cP and 354 mOsm, respectively. These parameters were not affected by the presence of SPIONs on the NB surface. Furthermore, OLNBs showed homogenous size and stability over 3 months.

**Figure 1:**
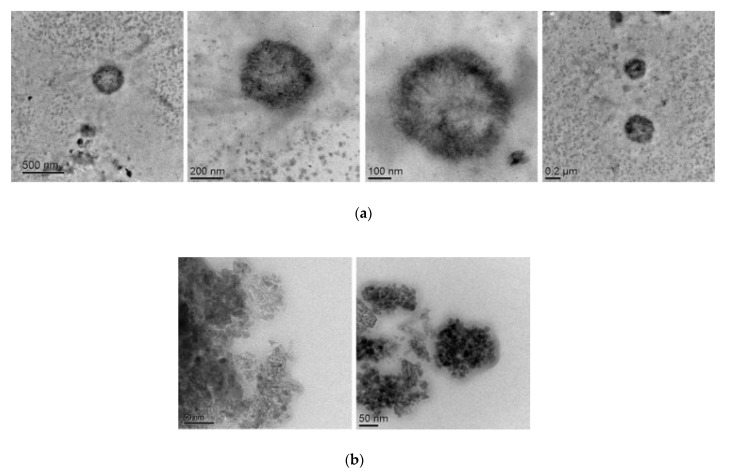
(**a**) TEM images of MOLNBs at different magnification. (**b**) TEM images of SPIONs.

TEM images both of SPIONs and MOLNBs were acquired at different magnification. The TEM images of MOLNBs showed a spherical and hollow morphology, with a well contrasted shell, as shown in Figure 1a. An average diameter of about 350 nm was detected for MOLNBs, confirming DLS measurements. The SPIONs structure is reported in Figure 1b, showing sizes ranging from 5 to 30 nm.

### 2.2. Hemolytic Activity

MOLNB aqueous suspensions tested at different dilution (1:10, 1:100, 1:200, 1:400 *v/v*) did not present significant hemolytic activity, showing no red blood cell damage in comparison with control. These data suggest that this nanoformulation is biocompatible and suitable for potential in vivo administration without any hemolytic problems.

### 2.3. Evaluation of MOLNBs Internalization and Toxicity

**Figure 2:**
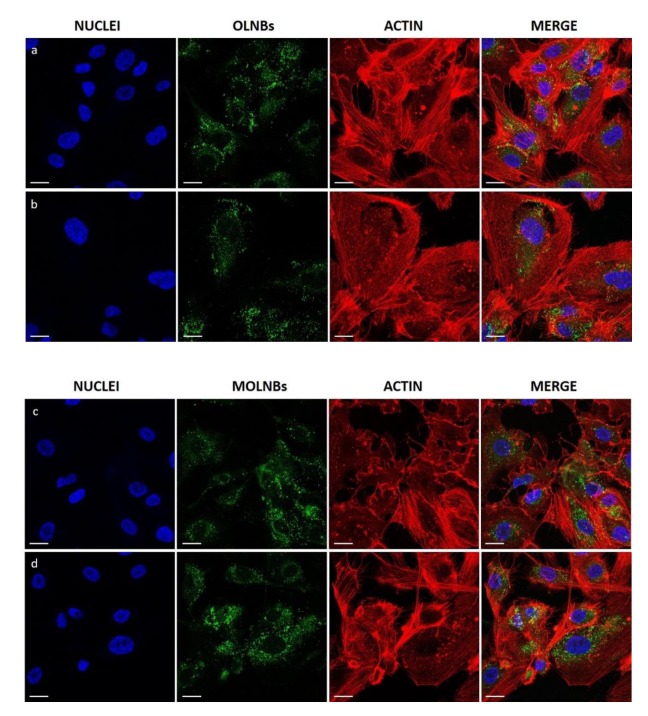
Confocal images of different formulation of nanocarriers internalized by hBMECs after 4 h of incubation. First and second rows: cells were treated with blank OLNBs (without SPIONs) in a dilution ratio 1:100 (**a**) and 1:200 (**b**) with the medium. Third and fourth rows: cells were treated with MOLNBs in a dilution ratio 1:100 (**c**) and 1:200 (**d**) with the medium. First Column: cell nuclei after DAPI staining, in blue. Second column: OLNBs and MOLNBs, conjugated with 6-Coumarine, in green. Third column: cell actin filaments after Rhodamine-Phalloidin staining, in red. Fourth column: merged images. Magnification: 40×. Calibration bar = 20 μm.

**Figure 3:**
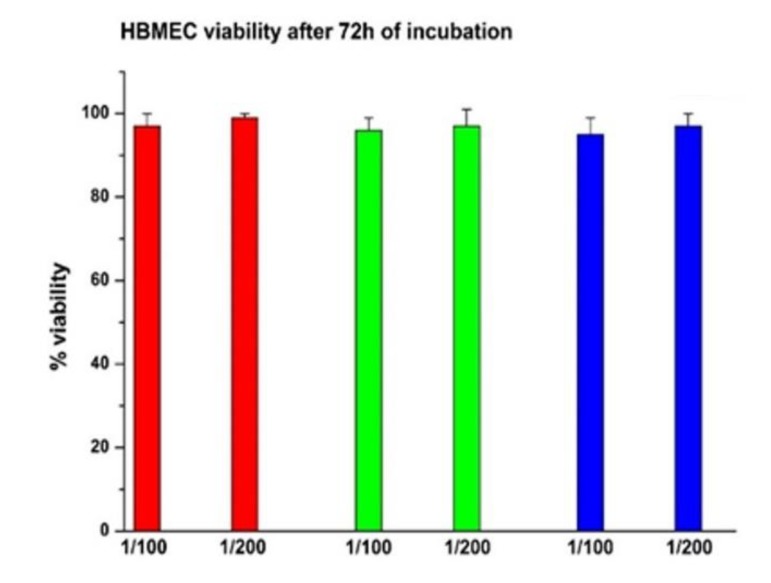
Percentage of viable cells after 72 h of incubation. The horizontal axis indicates the dilution (1:100 and 1:200) of NB. Red = MOLNBs; Green = SPIONs; Blue = OLNBs.

Confocal microscopy was used to verify whether MOLNBs were internalized by hBMECs. Images showed that MOLNBs, as well as OLNBs, were significantly internalized by hBMECs (Figure 2). Cytotoxicity studies underlined very good viability of hBMECs after the treatment with SPIONs, MOLNBs and OLNBs (Figure 3). 

### 2.4. US Monitoring of MOLNBs in the Magnetic Field

**Figure 4:**
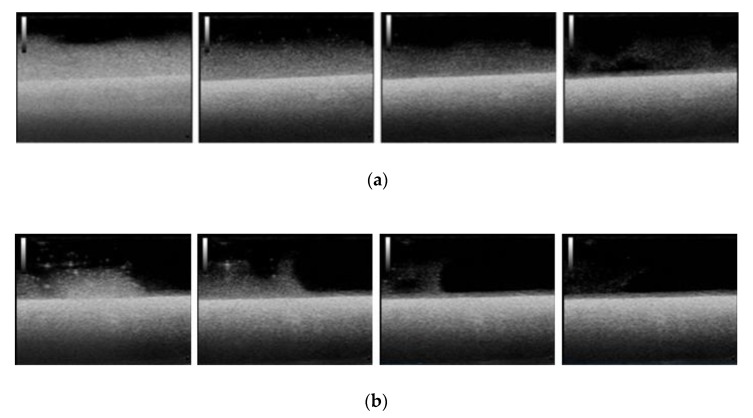
Snapshots from US imaging of MOLNBs in absence (**a**) and presence (**b**) of the magnetic field. Images were recorded at different time frames (5, 15, 25, 55 sec) from the injection.

The motion of the MOLNBs, located along the negative z-direction of the coordinate system, centered in the magnet, whose corresponding magnetization of the MOLNBs is dominated by the component along the *z*-axis, is significantly affected by the field. Snapshots at different time intervals from US imaging showed a different distribution of MOLNBs in the absence and in the presence of the magnetic field, which exerted a sensible effect driving a clearly detectable motion toward the magnet and along the axis (see Figure 4).

## 3. Discussion

MOLNBs are stable nanosystems showing a well-defined hollow structure, whose shell is densely decorated by SPIONs, as indicated by TEM analysis. The addition of SPIONs does not significantly increase neither their average diameter nor the polydispersity index, while the zeta potential is decreased but the value is still effective in preventing aggregation phenomena. This behavior suggested a good electrostatic interaction of SPIONs with the negatively charged NB shell. The combination of SPIONs with NB forming stable nanosystems was previously reported [20].

Furthermore, the surface negative charge makes MOLNBs promising candidates to efficiently cross the BBB, being the zeta potential a crucial parameter for brain delivery [21,22,23].

MOLNBs were able to store in the perfluorocarbon core and slowly release oxygen by passive diffusion gradient, as previously evaluated [5]. The oxygen concentration in the nanoformulation is related to the gas solubilization capability of perfluoropentane (80 mL O_2_ for 100 mL perfluoropentane). Indeed, the MOLNBs nanosuspension was saturated with O_2_ until reaching a 35 mg/L concentration in the aqueous media, during the preparation process. In these conditions, our previous study [5] showed a passive sustained and long-lasting oxygen release at different temperatures.

Interestingly, the versatile and peculiar architecture of MOLNBs can allow the incorporation of drugs with different chemical properties. In fact, the nanostructure consists of three domains: the core, the shell and the interface [24]. Their different lipophilicity was exploited for the incorporation of drugs, enabling high payloads [25,26,27]. Previously, chemotherapeutic drugs, such as doxorubicin and paclitaxel, were loaded in polymer-shelled NBs showing good stability and prolonged release kinetics [28,29,30]. Moreover, they were intravenously injected in mice without any acute side effects.

The absence of hemolytic activity of MOLNBs is an important aspect, being a key parameter for assessing the safety of the nanocarrier and biocompatibility, and is strictly required for intravenous administration in early preclinical development [31,32].

In vitro cytotoxicity assay and confocal microscopy images indicate that MOLNBs interact in a non-toxic way with hBMECs and are localized in the cytoplasm compartment of the cells, as result of internalization, even if the uptake mechanisms are still not completely elucidated. These results highlight the potentiality of MOLNBs to enter in the CNS cells. Furthermore, these data pave the way for future investigation concerning the ability of MOLNBs to cross brain barriers in an in vitro model.

The magnetic properties of MOLNBs were already investigated in previous research showing their superparamagnetic behavior and the possibility of monitoring their concentration in tissue with MRI [5]. In the same study we were able to prove that also US may be effective in detecting MOLNBs, due to their vaporizable perfluoropentane core and the density contrast of the SPIONs on their surface. It is worthy of note that perfluoropentane can undergo to Acoustic Droplet Vaporization (ADV) phenomenon, when irradiated by US [33]. The ADV favor the liquid to gas phase shift of perfluoropentane, increasing the US backscattering. The US imaging contrast ability of SPIONs decorated polymer nanobubbles was previously demonstrated by Luo et al. [34].

Therefore, we manufactured a simple device able to visualize the motion of the MOLNBs in a static magnetic field with field lines in the direction of injection by sonication. This preliminary magnetic investigation shows that MOLNBs can be magnetically guided using external permanent magnets. 

The above results support our initial speculation, since MOLNBs might be safely administrated either systemically or locally via intravertebral injection in the CSF, monitoring their concentration by MRI or sonography. Several studies evaluated the distribution of intrathecally injected nanoparticles within the CNS, assessing the good ability of administration route via CSF rather than systemic delivery [35,36]. Interestingly, future investigation will involve the study of MOLNBs stability in real matrix fluids, such as serum or CSF.

Furthermore, MOLNBs may be magnetically driven towards target membranes, for instance the one separating CSF and the interstitial fluid of the brain located in the choroid plexus in the brain ventricles to deliver oxygen and chemotherapy drugs to brain tumors.

Tailoring the driving magnetic field based on the position and dimension of the brain tumor and the brain membranes to be crossed is a goal particularly challenging. Further investigations are required to validate such application by computational models based on MRI or CT tumor images (in silico models) and finally on in vivo animal models.

## 4. Materials and Methods

All reagents were of analytical grade and obtained from Sigma-Aldrich (St. Louis, MO, USA) unless otherwise specified. Epikuron 200^®^ was kindly provided by Cargill. 

### 4.1. Synthesis of SPIONs

Fe_3_O_4_ nanoparticles were synthesized by a co-precipitation method. In a typical synthesis, 0.99 g of FeCl_2_ 4H_2_O and FeCl_3_ 6H_2_O was dissolved in 50 mL of degassed deionized distilled water in a conical flask and the temperature was moderately increased to 85 °C under continuous nitrogen purging and stirring at 700 rpm for 45 min. Then, 20 mL of ammonia solution 30% was added dropwise to the reaction mixture, and after pH reached 12 the reaction followed by stirring at 85 °C for 1 h. After the system reached a precipitation state, it was allowed for cooling and settling of the precipitation at the bottom of the flask at room temperature. The black colored precipitates were separated from the supernatant by using a permanent magnet. The precipitate was rinsed with distilled water and allowed to expose in air for 24 h.

### 4.2. Preparation of MOLNBs Formulations

**Figure 5:**
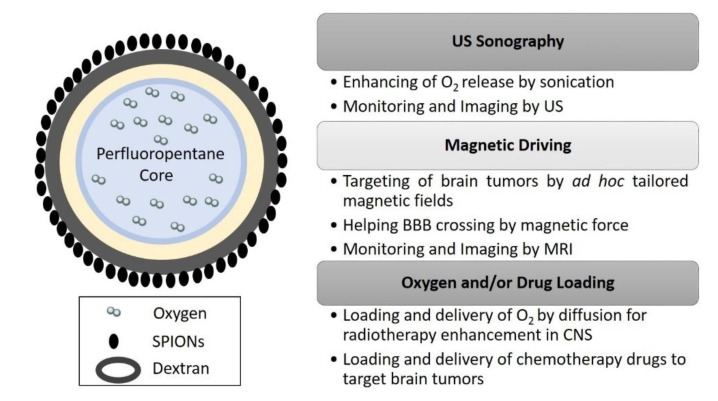
Sketch of MOLNBs (dextran NB covered with Fe_3_O_4_ nanoparticles, not to scale) and relative multifunctional applications as theranostic system in CNS.

Epikuron 200^®^ (2.5% *w/w*) and palmitic acid as a co-surfactant (0.5% *w/w*) were dissolved in ethanol. 300 µL of the mixture was added to perfluoropentane (PFP. C_5_F_12_) in an ice bath. Then the appropriate volume of distilled water was added dropwise to the mixture. The system was homogenized for 2 min by using a high-shear homogenizer (Ultra-Turrax^®^) until the formation of a nanoemulsion. Thereafter, the nanoemulsion was saturated with O_2_ for 10 min. Then, an aqueous solution of dextran sulfate sodium salt (2% *w/w*) was added dropwise to form the nanobubbles (NB) polymeric shell under an oxygen purge. Finally, 2 mg/mL of SPIONs suspension were added to NB under stirring, to obtain MOLNBs (see Figure 5).

For the fluorescent nanocarriers, 6-Coumarine (Sigma-Aldrich) was loaded into the MOLNBs core upon the addition of the fluorochrome directly to perfluoropentane solution. As a control, blank dextran shelled-OLNBs were also prepared, without the addition of SPIONs.

### 4.3. Physicochemical Characterization of MOLNBs

The mean hydrodynamic diameter, polydispersity index (PDI) and zeta potential of the SPIONs and MOLNBs were measured by dynamic light scattering spectroscopy (DLS) at room temperature. The samples were diluted with ultrapure water in an electrophoretic cell. Each measured value was the average of ten reciprocal, an electric field of 15 V/m was used for zeta potential determination. Photon correlation spectroscopy (PCS) with a scattering angle of 90, temperature of 25 °C using a 90 Plus instrument (Brookhaven, NY, USA) was used. The viscosity of the NBs formulation was determined at 25 °C using a capillary viscosimeter. The osmolarity was determined at 25 °C using an osmometer. The morphology of MOLNBs was observed by Transmission Electron Microscopy (TEM) in conventional mode, by using a JEOL2200FS microscope. The diluted NB aqueous suspensions were sprayed on the Formwar-coated copper grid and air-dried before observation.

### 4.4. Physical Stability of MOLNBs

The physical stability of blank OLNBs and MOLNBs was evaluated for up to 12 weeks. The parameters monitored for stability were average size distribution and surface morphology (shape of bubbles visualized by using microscopy) of the stored NB formulations at 4 °C. 

### 4.5. Determination of Hemolytic Activity

Hemolytic activity of MOLNBs was evaluated on rat blood. Blood was diluted 1:10 with PBS (pH 7.4). Triton × 1% was used as a positive control, where red blood cell breakage and further release of hemoglobin occurred. Saline solution (NaCl 0.9% *w/v*) was used as negative control. 1 mL of all samples were prepared (1:10, 1:100, 1:200, 1:400 dilution) and incubated for 90 min at 37 °C. Then, samples were centrifuged for 5 min at 2000 rpm and the supernatant analyzed with an ultraviolet-visible spectrophotometer (DU 730, Beckman Coulter, Fullerton, CA) at λ = 543 nm. The percentage of hemolysis was calculated using the positive control as 100% hemolysis. 

### 4.6. Evaluation of MOLNBs Internalization by Human Brain Microvascular Endothelial Cells

Human brain microvascular endothelial cells (hBMECs), provided from Cell Systems (Kirkland, WA, USA), were cultured in EndoGRO Complete Medium (Merck Millipore), plated in 24-well plates on glass coverslip (5 × 10^4^ cells per well) and incubated for 4h in a 500μL of medium with/without MOLNBs and OLNBs (dilution 1:100 and 1:200) internalized with 6-Coumarine (Sigma-Aldrich) in a humidified CO_2_/air-incubator at 37 °C. Fixing was carried out by adding 500 μL of cold paraformaldehyde (PFA, 4%) and by incubating for 15 min at room temperature and rinsing the excess PFA with sterile PBS. After fixing, 4′,6-diamidino-2-phenylindole (DAPI) and Rhodamine-Phalloidin (R415, Invitrogen™, Thermo Fisher Scientific, MA, USA) staining reactions were performed to label cells nuclei and the actin filaments. Fixed cells were kept at 4 °C for 24 h and fluorescence images were acquired by a confocal laser scanning microscope (LSM 900, Carl Zeiss, Oberkochen, Germany) equipped with a 40X oil immersion objective, obtaining a field view of at least 5 cells. A wavelength of 505 nm was used to detect MOLNBs and OLNBs, of 565 nm and 460 nm to detect respectively the actin filaments and the nuclei. Images were processed using the software ImageJ (https://imagej.nih.gov/ij/).

### 4.7. In Vitro Cytotoxicity Study

The hBMECs cells were used to perform in vitro cytotoxicity test. Cells (800/well) were seeded in 96-well plates and incubated at 37 °C, 5% CO_2_ for 24 h in EndoGRO Complete Medium. Then, the cells were treated with OLNBs, SPIONs, MOLNBs, in two different dilutions with medium (1:100 and 1:200). After 72 h incubation, viable cells were evaluated by 2,3-bis [2-methoxy-4-nitro-5sulphophenyl]-2Htetrazolium-5carboxanilide (MTT) inner salt reagent at 570 nm, as described by the manufacturer’s protocol. The control cells were normalized to 100%, and the readings from treated cells were expressed as percent of cell viability. Eight replicates were used to determine each data point and four different experiments were performed.

### 4.8. Magnetic Field

**Figure 6:**
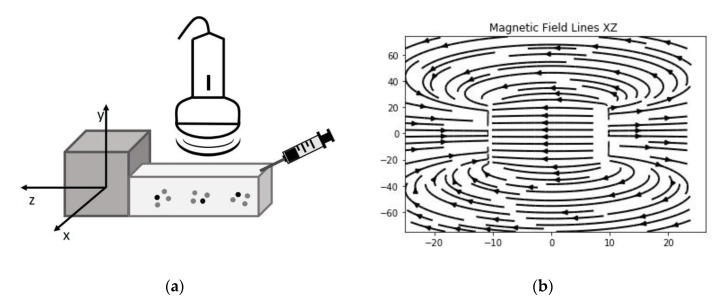
(**a**) A sketch of the setup used for the imaging of MOLNBs in absence and presence of the magnetic field produced by the cuboid magnet. (**b**) Projection of magnetic field lines in the XZ plane assessed by the z-direction of the magnetic field.

The small tank (see Figure 6), where the MOLNBs were sonicated, was positioned with its horizontal axis aligned with the axial field along z-direction generated by a permanent cuboid magnet of neodymium covered with Ni-Cu-Ni with dimensions 50 × 50 × 20 mm^3^ (https://calamite.org), inducing a field 1.26−1.29 T. The value of residual magnetization of the permanent magnet is in the range of 1.00−1.03 × 10^6^ A/m. The field lines were investigated using iron filings showing an intense magnetic induction almost parallel to the axial direction. Simulations of magnetic field lines were obtained using the analytical expression of the three-dimensional flux density distribution [13,37].

### 4.9. US Imaging Monitoring

B-mode US imaging was carried out to investigate the response of MOLNBs to the external magnetic field due to their excellent echogenicity [5]. MOLNBs at concentration 1 × 10^10^ NB/mL were injected in a plastic tank containing demineralized water by means of syringe positioned as indicated in Figure 6. The plastic tank was made by 3D printer to obtain the dimensions 7.5 × 2 × 3 cm^3^ to well fit with the probe dimension. A sketch of the setup is shown in Figure 6a. The experiment was performed at a temperature of 20 °C. MOLNBs were sonicated by an US clinical equipment (MyLab™25Gold Esaote, Genova, Italy), connected to a linear array transducer (LA523, 7.5 MHz central frequency, Esaote, Genova, Italy) operating in B-mode using the small parts imaging preset. B-mode cineloops (60 sec) were acquired and recorded for postproduction both in the absence and in the presence of the permanent magnet exerting a magnetic force in the direction of injection (see Figure 6b). Snapshots from cineloops were extracted at different time frames (5, 15, 25, 55 sec) after the initial injection and compared in the different conditions.

## 5. Conclusions

Our study provides a complete characterization of the physicochemical properties of MOLNBs and demonstrates their biocompatibility and safety in the case of systemic administration. In addition, this nanoformulation might be considered a good starting point for developing a system able to cross the brain membranes, main obstacle to enter CNS. Furthermore, our preliminary results highlighted the capability of MOLNBs to be magnetically driven. Summarizing, this work opens new opportunities to consider MOLNBs in targeting brain tumors since they can deliver oxygen (potentiating radiotherapy) and chemotherapy drugs, being driven by ad hoc tailored magnetic fields under MRI and/or US monitoring.

## Figures and Tables

**Table 1:** Physicochemical characteristics of different nanocarriers formulations. Data reported as Mean ± Standard Deviation.

Formulation	Average Diameter (nm)	Polydispersity Index	Zeta Potential (mV)
Blank OLNBs	331.6 ± 19.7	0.22 ± 0.10	−35.36 ± 4.16
Fluorescent OLNBs	338.2 ± 13.8	0.24 ± 0.08	−34.24 ± 6.52
MOLNBs	349.2 ± 18.2	0.21 ± 0.01	−20.41 ± 8.60
